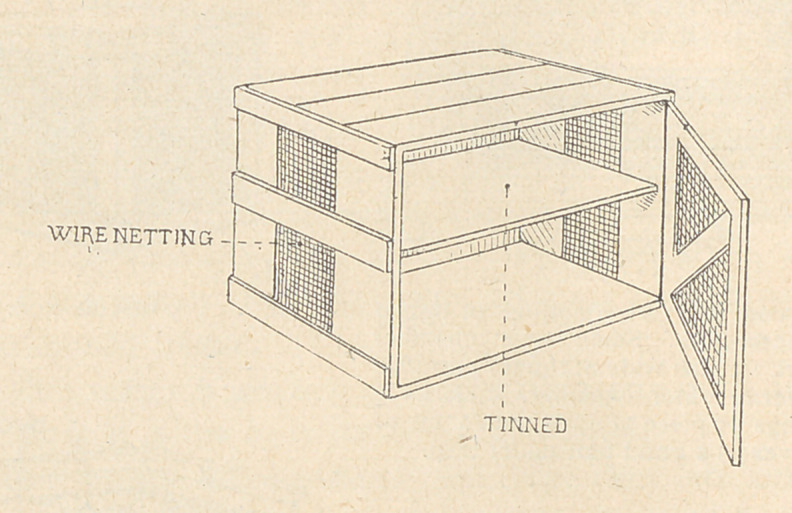# Circulars, Bulletins, and Reports from the C. S. O.

**Published:** 1918-10

**Authors:** 


### CIRCULARS, BULLETINS AND REPORTS

### Issued from the Office of the Chief Surgeon, of the American Expeditionary Forces in France.

*Under this heading will be published extracts from circulars and bulletins issued by the Chief Surgeon of the Medical Department of the American Expeditionary Forces in France. It is believed that these will be of general interest and value to medical officers.*

### EXTRACTS FROM C. S. O. CIRCULARS

### Papers for Publication.

The attention of all medical officers is called to the following memorandum which has been received from the Surgeon General. Papers for publication should be sent through the office of Chief Surgeon.

" Attention is called to the memorandum quoted below, which was issued March 27, 1918. In many instances paragraph three (3, has been overlooked. It is essential that this office receive *in duplicate* all professional papers submitted for authority to publish:

" 1. Attention of medical officers is directed to the provisions of paragraph 423 M. M. D. Medical officers will not publish professional papers requiring reference to official records or to experience gained in the discharge of their duties without the previous authority of the Surgeon General.

2. Numerous scientific papers written by officers of the Medical Department have recently appeared in the medical press without specific authority from this office. This practice will be discontinued and the above regulations will be strictly complied with.

3. Officers desiring the publication of professional papers will submit two copies to the Surgeon General with request for permission to publish same. Upon approval, a copy will be forwarded to the journal designated by the officer for publication. "

### Instructions concerning the treatment in orthopedic conditions Including fractures and joint injuries.

1. Upon the recommendation of the Chief Consultant in Surgery, and with the approval of the Director of Professional Services, the following instructions are published for the information and guidance of all concerned :

2. The work of the Division of Orthopedic Surgery in the medical organization of the army divides itself quite clearly into two parts, one having to do with the preparation of the men for the expected combat, and the other assisting in their recovery if wounded. The first endeavors to see that they are so trained that there will be the greatest possible vigor for the combat, and that physical defects which might have rendered them ineffective are corrected. The second has to do with the treatment of the men, if injured, so that there will be the least possible ultimate crippling or interference with function. The first has to do with saving men for service who would otherwise be discharged as physically unfit and also, as the result of careful training, increasing the number of days that should be expected of the men for active duty. The second has to do with the saving for service of men who but for such work might not have lived, or, had they lived, been so crippled as to be of no use to the Army.

3. Without such methods of treatment available for those needing such care in the pre-combat or training period, large numbers of men will be lost for active duty as the ordinary medical measures can only give temporary relief.

4. Without such methods in cases of combat or other injury there will be much unnecessary loss of function and much of the acute surgical treatment will be purposeless.

5. In each of the large hospital centers, a base hospital with special personnel and equipment for caring for such cases will be installed, while in the detached base hospitals special services will be established so that there will be the least possible transferring of cases from one hospital to another.

6. Consultants in Orthopedic Surgery will be assigned to groups of hospitals whose function it will be to keep in touch with the orthopedic work of the given group. These consultants should be freely used by the staff of the respective hospitals and can be reached through the commanding officers of hospital centers.

7. To best accomplish the purposes of the Division and to make the services of its members available, the following instructions will govern :

*Amputations.* 8. Cases of amputation of either extremity will be assigned as soon as possible to the orthopedic service for the needed special treatment. A guillotine amputation for instance, without other injuries, can usually be moved without risk in one week, and with suitable measures rapid closure of the wound is usually possible so that the artificial leg can be fitted, and many times the man can get about without crutches in from four to five weeks from the time of injury. It is desirable that transfer to the orthopedic service take place as early as possible before contractures have taken place, so that the temporary artificial limb, in case that is desirable, can be most favorably fitted and the muscles used to the best advantage,

*Tendon Injuries or Inflammations.* 9. The cases of injury to the tendons, or inflammation in or about the tendons, should be assigned as soon as the primary wound healing is well established, or as soon as the acute inflammatory reaction has subsided to the orthopedic service. Early transfer to these special services is important in order that the treatment having to do with the full restoration of function in the part that has been injured or inflamed may be established at the earliest possible moment, and before adhesions have formed or become organized.

*Flat feet, Weak Feet or Pronated Feet.* 10. Cases of flat, weak or pronated feet associated with pain, swelling or inflammation, when admitted to a hospital should be assigned to the orthopedic service. As soon as the acute symptoms have passed, the cases should be transferred to the nearest convalescent camp. From here, in keeping with the degree of difficulty, the cases should be transferred for full duty or to the Orthopedic Training Camp, Depot Division, for training to fully overcome the weakness, or for non-combat duty under Class "C" classification.

11. No cases of uncomplicated flat foot should be exempted from service or recommended for transfer to the U. S., as all can be made useful for military service.

*Spinal Strains, Weak Backs — Chronic Back-aches.* 12. The cases of weak, painful or lame backs, or of sprain of the spinal or sacro-iliac joints, should be assigned to the orthopedic service. From here they should be transferred to the nearest convalescent camp as soon as the acute symptoms have passed, and from there, after a reasonable time, they should be transferred either for full duty, or for non-combat duty under Class "C" classification.

*General Bad Posture.* 13. Cases of general bad posture, which is commonly associated with lack of vitality or general endurance, as well as being part of the condition leading to weak feet and weak backs, should be sent for training in the Orthopedic Training Camp, Depot Division.

*Fractures.* 14. For all cases of fracture of bones other than of the head or face, or of extensive muscle injuries, it is of the utmost importance that proper splints be applied at the earliest possible moment so that the transfer of the patients to the hospital in which treatment is to be given is associated with the least possible damage to the tissue adjacent to the injured bone. The

Thomas Leg Splint, the Hinged Half Ring Splint, the Thomas Hinged Arm Splint (Murray modification), the Cabot Posterior Splint and the Ladder Splinting are the appliances most needed for such work.

15. In case the fracture is compound, the wound treatment at the evacuation or other hospitals should follow the principles outlined by the Chief Consultant of Surgical Services.

16. After the primary wound treatment has been given, thes cases should be transferred to the orthopedic service in which th most approved methods for the early restoration of function to th injured part will be available. An effort should be made to transfer the cases to such services, wherever possible, within a week or ten days of the time of injury, this being the most favorable time as regards bone repair. All fracture cases which, for any reason, cannot or should not be transferred to one of the services as indicated above, should be reported to the Senior Consultant in Orthopedic Surgery, or to the Orthopedic Consultant of the special area.

17. Simple fractures should not be converted into open fractures except under very exceptional conditions or after consultation with one of the orthopedic consultants. A result which may not be as perfect anatomically as might have been obtained by open operation may, nevertheless, be functionally good. This is so commonly the case that the risk of infection which is greater under the war conditions than in civil life should be avoided when ever possible.

*Joint Injuries.* 18. All injuries of the joints should be protected with the same care for transport to the hospital in which the treatment is to be given as has been indicated for fractures. Suitable splints should be applied immediately and the standardized list of splints of the Army provides types that will meet all the needs.

19. In case the injury is associated with open wounds, the principles of wound treatment are those which have been laid down by the Chief Consultant of General Surgery.

20. Since in all such injuries ultimate function of the joint is the chief requisite, treatment having for its purpose the restoration of function should be instituted as soon as possible, and for this purpose it is desirable that cases of such injury be transferred, as soon as the primary wound treatment has been given, to the orthopedic service. It is important that such transfer be made before necessary adhesions have formed, so that the restoration of function can be obtained with the least possible loss of time. In all such functional restoration it should be clearly understood that while motion is to be encouraged at the earliest possible moment, it should consist entirely of active motions performed by the patient, in which case the reflex muscular contraction will protect the joint from undue injury. All passive motion should be avoided.

21. Operations upon the joints that are not emergency in character should not be performed until after consultation with one of the consultants in orthopedic surgery.

*Transfer to United States.* 22. It will be the policy to send to the United States, as soon as transportable, all cases that are of Class "D" type, or cases in which prolonged regiment will be required for restoration to duty.

### Collection of Museum Material for Medical Education and Research.

1. *Object.* This circular is for the information of these branches of the service whose co-operation and assistance are necessary to enable the Army Museum to discharge its duty of collecting all those things which may be used for medical education and research, or which may be of historic interest. This material will consist of pathologic specimens, bacteria, animal parasites, missiles, armor, instruments, apparatus, casts, models, paintings, drawings, diagrams, charts, statistical tables, cinema films, photographs, radiographs, lantern slides and other things pertaining to the preservation of the health and the prevention and treatment of the diseases of United States soldiers, or to the history of the Medical Department of the Army.

2. *Scope.* In France all collections will be limited to those things which cannot be obtained readily in the United States, or which are necessary for study in the A. E. F. More specifically these will relate principally to war wounds, especially lesions of bones and vital organs, gas poisoning, trench foot, gas gangrene, traumatic and "shell" shock, to infectious and parasitic diseases of special menace to the A. E. F. and to material of historic interest. Other material may be included if obviously desirable. It is requested that all medical officers in the A. E. F., cognizant of desirable museum material which they are not in position to direct into proper collection channels, should notify the Director of Laboratories, A. E. F. (Museum Unit) A. P. O. 721.

3. *Responsibility.* It is the duty of each medical officer in the A. E. F. to direct into proper channels all such desirable material coming to notice. In each medical unit the pathologist, or, in his absence, some other medical officer, will be responsible for the collection, preservation and shipment of all such material obtainable in the unit.

4. *Use in A. E. F.* Collected material required for investigation in the A. E. F. will be shipped as early and as directly as possible to the groups of officers conducting the investigations, in such manner and quantity as they may request through the Director of Laboratories, A. E. F. After serving the needs of the immediate investigation, this material, if still of value, will be preserved for use elsewhere.

Requests for material required for teaching in the A. E. F. should be made to the Director of Laboratories, A. E. F., who will direct from what source it shall be supplied.

5. *Concentration Points.* All other collected material will be shipped without unnecessary delay directly to concentration points as follows:

(*a*) To the Central Medical Department Laboratory from all hospitals in the Southeastern portion of the Zone of Advance and from other hospitals to which the Central Medical Department Laboratory is most readily accessible.

(*b*) To American Red Cross Military Hospital No. 2 from all hospitals in the middle section of the Zone of Advance to which it is most readily accessible.

(*c*) To U. S. Base Hospital No. 4 (B. E. F. No. 9, General Hospital) from all hospitals in the Northern portion of the Zone of Advance to which it is most readily accessible.

(*d*) To U. S. Base Laboratory of Base Section 1, or to U. S. Base Laboratory of Base Section 2, from all hospitals to which either of the above points is most readily acccessible.

The local Railway Transport Officer should be consulted as to the most accessible point for concentration of packages at the time shipment is to be made.

6. *Final Disposition.* At the concentration points the Museum Unit will take charge of the further preparation of all material and its shipment to the Army Medical Museum. There it will be cataloged and such portions of it as are necessary immediately redistributed as loans in accordance with a recent decision of the Surgeon General's Office, as follows:

(*a*) Teaching material to U. S. Army Schools for medical officers.

(*b*) Teaching and certain research material to the undergraduate medical schools of the United States (all of which are now under the supervision of the Surgeon General's Office).

(*c*) All historic and surplus material will be held in the Army Medical Museum for local use or future loans.

7. *Pathologic Specimens.* All pathologic specimens suggested in par. 2, from both operations and autopsies, should be preserved as follows :

(*a*) Gross Specimens. These should be dissected enough to disclose the character of the lesion and to permit proper fixation. The surface blood should be rapidly washed off with weak formalin (1 o/o or previously used). Each should have securely attached to it a tag of starched cloth or thick, tough paper on which is heavily written in black lead pencil or typewriting the name, rank and organization of the patient, the anatomical name of the specimen, the diagnosis of the lesion, the hospital number, the serial number of the specimen, (if autopsy material, the autopsy number) and the date of collection. Each specimen should be fixed, and preserved until shipped, in 5 to 10 times its volume of Kaiserling No. 1 solution, the formula of which is as follows :

| | |
|---|---|
| Potassium nitrate . . . . . . . . . . . . | 15 grams. |
| Potassium acetate . . . . . . . . . . . . | 30 " |
| Formalin . . . . . . . . . . . . . . . . . . | 200 c.c. |
| Water . . . . . . . . . . . . . . . . . . . . | 1000 c.c. |

These materials may be requisitioned.

Sodium salts may be used instead of potassium. If materials for other methods of color preservation are at hand, they may be used, but the specimens kept separate from others in shipping. If no salts are obtainable 10 o/o formalin may be used. Hollow organs, lungs, intestines, etc., should be filled with the solution to their normal size and caliber. Where time permits, the vessels of large specimens should be injected with the solution.

The solution fixes very rapidly and rigidly so that it is necessary to use care when specimens are placed in it that they are not deformed by pressure. Soft organs, (brains, lungs, etc.) which may be injured by pressure, should be fixed in individual containers, (jars, granite-ware pails or pans, kegs, etc.). Other tissues may be fixed several together in tubs, barrels, casks, etc.

Specimens should not be placed in containers in contact with metal nor in new wooden vessels the walls of which may contain tannin. If new wooden vessels are used they should be coated inside with paraffin. Large containers— earthenware jars, barrels, casks, etc. — should be obtained locally. Wide-mouth bottles and small specimen jars may be obtained by requisition.

After preliminary fixation the specimen should be changed at least once to fresh fluid which may be reduced in strength to 10 o/o formalin. Delicate specimens, such as pieces of intestine or blood vessels, need to be carried through the entire Kaiserling process rapidly if a brilliant color is to be preserved. With all other specimens only the Number 1 need be used.

Where the specimen is a bone the soft parts should be left attached and the specimen treated similarly to lesions of soft tissues alone.

(*b*) Material for Microscopic Examination. Tissues intended especially for microscopic examination should be cut with a sharp knife or razor into thin blocks (not over 0.5 cm. thick) and placed immediately into 20 to 50 times their volume of fixative. (Zenker's fluid, formol-Zenker, neutral Zenker, 10 o/o formalin, 95 o/o alcohol, or other). Their source should be accurately noted, described and sketched. Their subsequent treatment should be that appropriate for the fixative. Special attention is called to the necessity for fixing tissues intended for cytologic study as soon as possible (under two hours) after circulation in the part has ceased. Wide mouthed bottles or small glass jars tightly closed should be used as containers for histologic material.

8. *Shipment.* When pathologic specimens have been fixed for two weeks or more they should be well padded with absorbent cotton wetted with the solution in which they have been last immersed, then wrapped in water proof paper (to be obtained by requisition) and packed with paper, excelsior, hay or similar material in a strong wooden or tin box or a barrel and shipped to the most accessible point of concentration. (See paragraphs 5 and 6). Each package should be marked with the hospital number, the serial numbers of the specimens, the autopsy number, if any, and date of shipment.

At the same time there should be forwarded by mail or courier an inventory of the contents of each package accompanied by abstracts of the clinical records of operation specimen, and of clinical and autopsy records of autopsy specimens. The name of the pathologist or other medical officer who may be specially interested in the specimen should be given.

Army Regulations authorize transportation of all museum material by the Quartermaster Corps. Packages of specimens weighing 7 lbs., or less, should be directed on a penalty envelope, marked official, and delivered to an A. P. O. of the Military Postal Express Service with explanations of their character and the importance of their prompt delivery to prevent spoiling.

9. *Bacteria.* Army Regulations provide that cultures of all pathogenic bacteria isolated in the A. E. F. shall be sent to the Central Medical Department Laboratory for confirmatory identification. The museum supply will therefore be drawn from the Central Medical Department Laboratory.

10. *Microscopic Slides.* Microscopic slides containing data which cannot readily be duplicated in other material sent from the same source should be sent to the appropriate concentration point.

11. *Animal Parasites.* Specimens of animal parasites — if possible living — such as lice, fleas, mites, bugs, flies, mosquitoes, worms, etc., should be sent to the Central Medical Department Laboratory for confirmatory identification. The museum supply will be drawn from this concentration point.

12. *Missiles.* For the psychic effect a missile removed from the body of a wounded soldier may be given to him if he wishes to keep it. However, he *may* be induced to relinquish his claim when the scientific value of the comparative study of such missiles and their preservation in a museum is explained to him. The place and character of all missiles in amputation material should at least be accurately described, and, if possible, sketched. All missiles and foreign bodies removed at autopsies should be carefully preserved, if possible in situ, with the pathologic specimen. When it is necessary to remove them, their location and wound effects should be minutely described, the description, if possible, being accompanied by photographs or sketches.

13. *Armor.* Armor, such as helmets, or other protective body covering showing the effects of missiles, gases, etc., should, whenever obtainable, be preserved with full data concerning the incidents of their use, and shipped to the nearest concentration point.

14. *Instruments and Apparatus.* All instruments and apparatus of special value, which have been developed or materially modified in the A. E. F., should be photographed, accurately described, and, if it seems desirable, models made and sent to the nearest concentration point.

15. *Cast and Models.* The number of skilled cast and model makers in the A. E. F. is extremely limited. When a medical officer has some specimen, or series of specimens or cases, showing results of operations which he wishes to have illustrated in wax or plaster, he should make application to the Director of Laboratories, A. E. F., (Museum Unit), A. P. O. 721, for the services of a model maker.

16. *Paintings, Drawings, Diagrams, etc.* It is believed that in many hospital units there may be found men capable of making diagrams and sketches furnishing graphic records of teaching or historic value to the Medical Department. Well trained medical illustrators, on the other hand, are scarce and their services, to be utilized in an economical manner, must be centrally controlled. Medical officers having material of scientific value, particulary in the fields noted in paragraph two, and who are without the assistance of capable medical illustrators in their hospital units, should apply to the Director of Laboratories, A. E. F., (Museum Unit), A. P. O. 721, to have an artist assigned for temporary duty.

17. *Cinema Films.* There are a few subjects, — e. g., patients with " shell " shock, the technique of new operations, etc., — records of which it may be desirable to preserve in moving picture films. Application for the services of a cinema camerist for this work should be made to the Director of Laboratories, A. E. F., (Museum Unit), A. P. O. 721.

18. *Photographs.* General Order 78, G. H. Q., A. E. F., May 25, 1918, amends previous orders as follows : " The Medical Department, A. E. F., is charged with technical photography connected with the recording of photographic processes of surgical and pathological matters ". For the proper discharge of this duty each hospital unit should have on its personnel, either in the laboratory or roentgenographic department, at least one man capable of taking good technical photographs of medical subjects. A standard laboratory photographic outfit should be requisitioned by each Base Hospital not already equipped. It is assumed that all developing will be done in the X-ray darkroom where will be available a ruby light, and all necessary chemicals for development and fixation of plates and prints.

Photographic records should be made of interesting lesions, particularly in the fields noted in paragraph 2, and of those things of medical, surgical, or pathological interest in the hospital which may be of value for teaching, research or for their historical connection. Copies of these should be forwarded by mail or courier to the Central Medical Department Laboratory, (Museum Unit), A. P. O. 721, as soon as made, and the negatives reserved for subsequent shipment to the most accessible concentration point.

18. *Radiographs.* Radiographs, especially those in series or illustrating wound conditions or their treatment which may be of value for teaching, should be copied in prints or lantern slides, which should be forwarded by mail or courier with full data to the Central Medical Department Laboratory, (Museum Unit), A. P. O. 721.

19. *Original Publication.* All pathological specimens, casts, models, paint ings, drawings, photographs, radiograms, etc., should be accompanied by the name of the medical officer conducting them, and of the medical officer, if any, specifically interested in their subject matter. This is important, not only for the occasional necessity for retracing them back to their origin for additional data, but also that the privilege of original publication of the data by the officer with whom they originated may be respected.

20. *Supplies.* All requisitions for supplies will be prepared and forwarded by the Medical Supply Officer of the hospital unit. Requisitions for laboratory supplies only will be made in quadruplicate, one copy being retained and three copies forwarded to the Director of the Division of Laboratories and Infectious Diseases, Office of the Chief Surgeon, A. P. O. 721, and it is desired that, as far as possible, requisitions be timed so as to permit shipment thereupon to be included in larger shipments from supply depots on ordinary requisitions. These special requisitions, therefore, should be sent approximately ten days prior to larger requisitions contemplated and should bear notation that shipment should be held pending the receipt of requisition of general supplies,

---

### EXTRACTS FROM WEEKLY BULLETIN OF DISEASE

### Orders Regarding Drinking Water. From Headquarters of a Base Section.

Hereafter all water for drinking purposes, that is not obtained directly from sources approved by the Chief Surgeon of this Base Section, shall be treated with calcium hypochlorite in the proportion of at least one standard tube of the chemical to not over forty gallons of water.

The hypochlorite shall be added in the manner specified by Circular N° 27, Office of Chief Surgeon, A. E. F.

Whenever water obtained from an approved source is placed in a Lyster bag or other container for storage, it shall be treated in the same manner as water from an unapproved source.

When it is impossible to obtain hypochlorite, all drinking water will be boiled at least twenty minutes.

The use of common drinking cups will be discontinued. Care will be taken that men do not drink directly from the spigots of Lyster bags.

### The Duties of a Battalion medical officer.

First : *Medical.* A mere doctor is a small man in the army. Pills are not important; commonsense and all the qualities of a man are. He must be a medical officer and must act for the benefit of the State as well as the patient, and differentiate between a slacker and a sick man. In order to do this he must know the man, and in order to know him he must get his confidence and be in sympathy with him. Second : *Sanitary.* In teaching sanitation we must remember a regimental medical officer must teach it to every man in the battalion, from the commanding officer down to the last private, and in order to enlist interest in sanitation intelligent men should be given a reason for rules. Third : *Constructive.* The medical officer must provide the place and facilities for the care of the sick and wounded; Fourth : *Supervision.* The regimental medical officer is the show horse by which the corps is judged. He must be tactful, with ability, and enjoy good fellowship with men without losing his personal dignity. He must be an example to his men, honorable, brave and upright. Above all he should be sympathetic and secure the full confidence of the men. He should be a leader in the social, moral and military hierarchy.

### Sanitary Details.

*A. General Order Issued From the Headquarters.* Base Section 2 directs Camp Commanders in the Section to furnish daily sanitary details to the Camp Surgeon in the following numbers in proportion to the strength of the Command :

| | | |
|---|---|---|
| Up to 1,000 | — 2 0/0 | of entire Command |
| 1,000-5,000 | — 1.1/2 0/0 | " " |
| 5,000-10,000 | — 1 0/0 | " " |
| 10,000 and over | — 1/2 0/0 | " " |

For all other troops and detachments two men per organization will be furnished.

This provision should enable camp surgeons of that section to control fly-breeding and feces disposal. Fly carriage of feces seems to have been responsible for the spread of dysentery at various ports in Base Section 2.

### Prevention of Respiratory Diseases.

Sanitary report from England, " Respiratory diseases, colds and bronchitis, have prevailed during June because of damp weather and soggy ground." Sanitary report from the south of France, "Respiratory affections common because of the extreme dryness and dust throughout the month."

Has the man and his habits nothing to do with the catching of colds and the acquisition of respiratory infections. Seek the common factor elsewhere than in climate. Is not human contact, that is, sneezing, coughing, spitting in close quarters, at least in part responsible. The weather is hard to control, but men's habits and surroundings can be modified by *Education and Action.*

*A Word to the Wise* (*Sanitary Inspectors*). Just ask a few men with one gold chevron on their left sleeves, or if you can catch a man with two, ask him how he dried his shoes, his coat, nay his very undershirt last winter. Certainly not by hanging it on a fence in the sun. The fences were frigid and the sun went altogether out of the heating and drying business from October to April. Slush, sleet, continuous drizzle and mist kept the A. E. F. wet, day and night, and *cold.* Please sit up and take notice and get drying rooms, closets, boxes, any old place near a stove pipe or an incinerator ready for wet weather. We lost many a splendid fellow from pneumonia and empyema last winter and had our hospitals full of sick because men had to sleep often in wet clothes and shoes. A pair of pipe pliers is an awkward tool to put a pair of frozen boots on with. The newly arriving divisions and those who see this land in sunshine are too easy with themselves when they send in sanitary reports claiming adequate facilities for drying clothes, "lines between barracks", "fenches behind the kitchen". In the long weeks ahead when France goes through the black cold tunnel of winter, provision must be made for drying clothes day and night.

Also, bathing in the river and washing socks by the brookside have no attraction in December, and the man who takes a hot bath under orders once a week whether he needs it or not must have his water indoors.

### Standard Preventive Measures.

As a perpetual precaution against many infections beside typhoid fever, the following note from the 1st Depot Division is well worth following. Much influenza and kindred infections would be prevented if messkits were thus sterilized: "Heretofore, messkits were being washed in two large containers of water, one soapy and the other clear. Repeated bacteriological examinations revealed the fact that this soapy water after its use was nearly sterile, while the latter, or clear water, showed 5 or 6,000 colonies per c.c. So the following plan was adopted in each kitchen : First, one large container filled with a hot, strong, soapy solution of clean water; Second, a large container of hot, clear water; and Third, one container was placed over fire and kept boiling while the men were using it. "

*Carrier Rate among Meningitis Contacts in the... Division* : Fifty cases of cerebrospinal meningitis in the division since Aug. 1917. All carriers left in U. S. when the division "came over", after culture of the entire command. Carrier rate before leaving U. S. 1.5 o/o-2 o/o, two cases of meningitis July 16 and 17, 1918, in one Infantry regiment, and two more on July 19 and 22 in another Infantry regiment. Seventy-three contacts of the above four recent cases thoroughly and repeatedly (3 times) cultured July 23-Aug. 2, 1918, and plates prepared in the spot and carried warmed to the Central Medical Laboratory where they were put in incubator within one hour after swabbing. *All contacts found negative for meningococci.* Such briefly is the history of a divisional problem and the not unexpected result of a midsummer culturing of noses and throats of men living an out-door life. If we had as much fresh air in winter, and treated acute colds as if they were serious communicable diseases, we should have similar though probably not such a 100 o/o result.

### Meningococcus Serum.

Bulletin of Diseases N° 14 contains the following sentence : " If the meningococcus is not found on smear or culture, it is well to be satisfied with withdrawal of fluid and not to give serum ". In a certain number of cases of epidemic meningitis, the meningococcus is not found on smear. Cultures take at least 24 hours and cannot be waited for, as early treatment is a most important consideration. The sentence in question should read : " Serum should not be administered unless a purulent fluid, or a fluid showing marked increase in leucocytes is withdrawn ".

Osler contains the following sentence : " Whenever the fluid obtained by lumbar puncture is purulent the serum should be given, but repeated only if the meningococcus is found ".

### Anthrax Controlled.

Measures taken in the United States to prevent the manufacture and distribution of shaving brushes made from anthrax infected bristles and hair appear to have been adequate. There were reported in March, 2 cases of anthrax; in April, 5; May, 8; up to June 17th, 8 cases; June 17th to July 21st, no cases.

### Facts About Trench Fever.

Prevention now entirely practicable upon the basis of scientific proof of source of virus and means of transmission.

The following conclusions have been officially endorsed by the committee of medical officers of the British and American Expeditionary Forces called to consider the results of the experimental clinical and laboratory investigation carried out upon the subject of Trench Fever. The clinical subjects were all volunteers from the enlisted men of the Sanitary Corps of the Medical Department, U. S. A.

1. The organism causing trench fever is a resistant filterable virus.

2. The virus causing the disease is present not only in the plasma of the blood but in the urine, and sometimes in the saliva or sputum of trench fever cases.

3. The disease is transmitted naturally and commonly by the louse Pediculus humanus, variety corporis; the louse may transmit the disease by its bit alone, or the disease may be produced artificially by scarifying the skin and inoculating the scarified area with a small amount of the infected louse excrement.

4. The louse need only remain upon an individual for a short period of time in order to infect him with trench fever, and a man may be entirely free from lice at the time he develops symptoms of the disease.

A series of inoculations relating to the thermal dead point and the resistance of the virus of trench fever have been made and will be reported upon as soon as the work is completed.

### Typhus Fever.

A case of typhus fever has occurred in the Advanced Section according to the best opinion of the medical consultants. The patient is from Battery "B", 305th Field Artillery organization. He left his former station at Camp de Souges in the vicinity of Bordeaux on July 5th, where he had been for two months, and during this time was occasionally in contact with Indo-Chinese troops. He first fell sick on July 12th, two days after his arrival. At the time of writing, the fever on the 13th day of the disease is still up. The incubation period of the disease — 12 to 14 days — would make the time and place of infection about July 1st at Camp de Souges. The necessary and very simple measure of protection by de-lousing was taken at the front area and at the base port. No secondary or suspicious cases have so far been reported. It is well to bear in mind that typhus has within the last two months been reported from Portugal, and that there is a constant marine commerce through the southern French ports now included in Base Section 7 with countries and peoples among whom typhus is endemic. The season of the year makes the likelihood of its spreading remote. There are some typical features in this case, viz : (1) Large erythematous plaques on the palms and on the soles of the feet: (2) A marked leucocytosis: (3) Lack of mental symptoms. On the other hand, the rash which appeared on the 4th day of the disease and developed upon the trunk seemed characteristic. The temperature has continued high. For sanitary purposes the case will be considered true typhus exanthematicus. *Therefore*, to " Kill the Cootie " is one way of clearing the road to Berlin.

" Whenever a contagious case appears in the command, the Division Epidemiologist is notified. He proceeds at once to this organization and assumes personal charge. He traces the source of contagion, isolates contacts, establishes and releases all quarantines."

" The clothing of all cases of pediculosis and scabies is sterilized at the de-lousing stations by the Sanitary Squads. The clothing of 716 soldiers and 80 prisoners of war had such treatment during the month."

" All troops arriving and departing from this command are examined for venereal diseases, vermin, cleanliness, etc. There are 52 portable bath houses in the division. All men are required to bathe at least once a week. Each bath house is in charge of an orderly from the Sanitary Squad who is unfit for heavier duty. This orderly keeps a roster of all men bathed so that the weekly consolidated report shows all men bathed at least once a week, except, for instance, at the Classification Camp. A large bath house at the latter camp is being constructed so that every man will be bathed and given clean clothing the day he arrives."

### Dysentery and Typhoid Diseases of Filth and Personal Carelessness

An army, even though vaccinated against typhoid and provided with Lister bags, can not afford to neglect the simple principles of personal cleanliness.

Look at the figures and be warned in time. All the communicable diseases, except those of the enteric group, have reached their lowest level for the year 1918 the past week.

| | | | | | |
|---|---|---|---|---|---|
| Diphtheria | cases per 100,000 strength | | | . . | 3.90 |
| Measles. | " | " | " | . . . | 3.90 |
| Scarlet Fever | " | " | " | . . . | 1.95 |
| Cerebrospinal Meningitis | " | " | " | . . . | 0.58 |

In the same week there has been a sudden and striking rise in the incidence of the intestinal infections — Typhoid and Dysentery cases per 100,000 strength 6.04.

Not alarming, but a direct proof, if any were needed, that the men of the A. E. F. are disregarding orders by drinking polluted water without chlorination or boiling, are eating food contaminated by the unwashed hands of cooks or kitchen help, and are exposing feces of carriers or cases of the enteric diseases to flies and human contact, through clothes, shoes and hands during the process of collection and disposal. From November 1917 to June 30, 1918 ,there were 36 cases of dysentery and typhoid, the dysentery in all but one

Trench latrine cover, squat type, hinged lids, automatic back-stop.

instance amebic and known to be recrudescences of long standing infections, and both diseases appearing simply and in widely separated commands. From July 1-21, 1918, there have been already imported 52 cases of typhoid representing two (2) distinct epidemic groups and 00 cases of dysentery, a majority being of bacillary origin, and 13 being in one focus.

Vaccination against typhoid and paratyphoid, however thorough, will not protect a command against massive infection due to conveyance of feces pollution direct to food or water. Artificial or natural immunity will break down under heavy infection.

*To be done now.* 1. Look over Records of Vaccination and where necessary revaccinate.

2. See that all water sources are labelled safe or dangerous, and the latter chlorinated before use. That means test water supposed to be chlorinated in Lister bags or water carts frequently to insure chlorination without excess or offensive dosing. Demand such policing of water sources, carts, and bags as to make the control of drinking water real and not merely theoretical. Water out of a brown canvas bag has no particular merit unless hypochlorite has been added as ordered. Sucking the nipples of a Lister bag in the absence of a cup is poor, however clean the sucker may think his mouth is.

3. Put feces out of reach of flies, food, and water as promptly as possible. It takes brains as well as G. I. cans to do this.

4. Demand clean hands and short finger nails and clean outer clothing of cooks and those who serve food. Have a basin and soap and water outside the kitchen and insist upon hand scrubbing before entering kitchen, especially after a trip to the latrine.

To quote from a valuable Bulletin on dysentery issued to medical officers in Base Section 2 on July 1st, " Every case of acute diarrhoea with pus or blood in the stools should be regarded as dysentery until proved otherwise. Before the specimen of stool starts for laboratory the diagnosis must be made, treatment instituted, measures to prevent spread taken, patient screened, stools disinfected. A medical officer will be judged by his morbidity rather than by his mortality rate. "

The epidemic of typhoid in the Camp Cody Replacement Detachment Co. apparently had its origin before arrival in the A. E. F. All the men have completed vaccination records. The epidemic of dysentery in the 36th aero squadron was due to the Flexner bacillus. The importance of the subject of the dysenterics can be measured by the following extracts from French and German medical publications.

### Bacteriological diagnosis unsatisfactory.

" The bacteriological diagnosis of dysentery during the war has been unsatisfactory; numerous epidemics, large or small, have been reported in which the bacteriological examination of the feces has failed to be of any help at all or has revealed dysentery bacilli in a very small percentage. Thus Dorendorf and Molle were able to isolate the Shiga-Kruse bacillus in 6 cases only out of 1,000 stools containing mucus examined in an epidemic in Galicia. In the second half of 1916 Schweriner made an extensive investigation on this subject and obtained a positive result in 18 per cent. of the cases examined. Dysentery bacilli are rapidly overgrown by saprophytes and so examination of the stools becomes progressively negative as time goes on; thus in cases examined within the first four days of disease 57 per cent. were positive, between the fourth and twelfth days 28 per cent., and in cases examined later 12 per cent. This bacterial overcrowding is favored by heat and can be considerably retarded by packing in ice the feces sent for examination."

"A camp epidemic of bacillary dysentery was investigated by Lesieur, Pellagot and Jacquet in the VIII military area of France which had been free from the disease since the summer of 1916, when there were 78 cases with 2 deaths at Bourges. The evidence showed that the epidemic was not due to contamination of the food or water supply, but that it depended on defective arrangements in the removal and emptying of pails containing the dejecta. The latrines belonging to the conscripts of 1918 were also used by some dysentery carriers and in this way the contents of the pails were able to spread the disease. In the previous epidemic ot 1916, reported by Durand, the spread of the disease also appeared to be due to a defect in the arrangements of emptying the pails of the latrines. When proper methods of collecting, emptying, disinfecting and cleaning the pails were established the epidemic of 1917 came to an end."

"In the epidemic of bacillary dysentery at Brest in 1916, investigated by Lancelin and Rideau, the majority of the cases were due to infection with Flexner's bacillus and were less severe and less toxic than those due to infection with the Shiga-Kruse bacillus, but, on the other hand, the Flexner bacillus cases were unfortunately prone to become persistent and chronic."

### Dysentery Widely Prevalent in Germany.

"On account of the prevalence of intestinal catarrh in Berlin, Schwee made inquiries from various medical men as to the incidence of the disease in Germany. The replies showed that dysentery was widely prevalent, especially in the Western and Eastern districts. In many cases Hiss's Y bacillus and in rare instances the Shiga-Kruse bacillus were cultivated. Simple, usually apyrexial, catarrh occurred mainly in North Germany, but was also present in the South, both in the civilian and military populations. By some it was ascribed to the usual dietetic factors and the summer heat, by others to the bad effect of war food, especially the bread. According to Strauss, of Berlin, most of these cases are due to an attenuated dysentery or paratyphoid infection, which are related to the war in two ways: (a) through the numerous carriers of these infections returned home from the front; (b) from the diminished resistance of infection due to mal-nutrition and the irritation of the intestine by coarse food. Most of the outbreaks may be regarded as connected with the war, especially from the greater consumption of indigestible or decomposed food."

*Act on Clinical Diagnosis*: Many medical officers are awaiting a laboratory diagnosis of specific bacillary dysentery before taking any special precaution to avoid spread or communication of the disease in the command. The warning and advice in Weekly Bulletin No. 15 should be followed in every instance. "Every case of acute diarrhea with pus or blood in the stools should be regarded as dysentery until proved otherwise. Before the specimen of stool starts for the laboratory the diagnosis must be made, treatment instituted, measures to prevent spread taken, patient screened, stools disinfected." The incubation period of bacillary dysentery is often as short as two days and rarely over four days. It takes at least four days for the laboratory to make a complete identification of the organism, during which time a whole new generation of cases may readily have developed unless instant action follows prompt clinical diagnosis.

### Cultures from Enteric Cases.

*To commanding officers of hospitals,* and for the attention of bacteriologists. A culture of the etiological organism isolated from cases of typhoid, paratyphoid A or B, bacillary dysenteries, or patients of the enteric group considered clinically as cases of the above named diseases should be sent by the bacteriologists of hospitals to the nearest Base Laboratory, or, if more convenient, to the Central Medical Department Laboratory, for verification, study and reference. The cultures should be sent as promptly as practicable, and by courier, if possible.

### Food Topics.

In spite of the routine and very rosy picture of food preparation and supply painted by the sanitary inspectors in their monthly reports, the following description is nearer the truth and suggests the need of a bit more imagination on the part of medical and line officers. In spite of the ubiquitous motor truck and the occasional passenger car, the army still walks on its belly. Improve the walking and quicken the pace.

### Messing Conditions.

There has been almost everywhere a distinct lessening of attention to the mess on the part of company officers compared to what existed in the States before the troops came over here. This is due in part to the conditions under which the troops live, especially in the billeted areas; in part to the intensive training of the officers in the technical side of the their work, and lack of time or attention to mess duties. The lack of accountability from the issue of the ration in kind has also had its effect in this direction.

Common defects are : (*a*) lack of attention to mess accounting and failure to attempt any saving at all; (*b*) lack of discipline in the kitchens, carelessness about cleanliness, lack of menu posting and checking by competent officers, lack of balance in menus and conspicuous lack of fruits or desserts, tendency to repeat standard stews and heavy dishes, failure to observe the value of palatability in menu and service, waste of food, large garbage waste, potato peeling everywhere, waste of fuel in many places, little use made of bones or soup, few renderings of fat on a scale suitable to get most out of the ration, lack of initiative on the part of cooks and mess sergents, no cook uniforms, etc., etc.

INEFFICIENCY AND WASTEFULNESS ON THE PART OF COMPANY COOK.

Under this heading should properly be included the almost universal practice of using lard and bacon grease as fuel rather than as food. Cooks have informed me that this is required by the absence of sufficient fuel; the dirty and unsanitary condition of many kitchens; the remarkable lack of interest in food conditions and their improvement on the part of company commanders. Every possible effort should be made to arouse an interest in the improvement of kitchen administration and sanitation by company commanders. A division bulletin calling their attention to the military importance of these matters would seem desirable.

REDUCTION OF WASTE IN THE MESSES.

1. Scrape or boil, and then peal, potatoes to avoid waste by paring.

2. Use up left-over potatoes in hashes.

3. Cut bread in fairly thin slices and cut the seconds in half slices. Allow the men to repeat as often as they desire. Frequently, however, on second helping a man wishes only half a slice of bread, and if he must take a whole one, the balance is wasted.

4. Hang the waste bread in a flour sack. It will not mould and as it dries out the dried bread can be used for many purposes beside bread pudding. For example, mixed with salmon for croquettes, mixed with butter for hot cakes, thickening gravies, bases of various forms of desert, with consequent saving of flour, etc.

5. The bean component adds a vegetable if properly flavored with bacon.

6. With the amount of meat and bacon issued the cooks should be able to secure by rendering a sufficient quantity of dripping to do all their frying and even a large portion of their pastry. In fact the lard component should be saved.

7. In serving food on the mess kits, it is very important to see that unappetizing messes are not piled on the mess kits. Often good food is spoiled and made repulsive to men by this action alone, with consequent waste.

8. Men will eat soups frequently and like them if they are well seasoned, thickened, and toasted bread croutons served in them. This use of soup means saving of the bone marrow, an increase in the bulk of the ration with reduction of meat consumption, and a way to use up old bread.

9. See that water is always available, for many men drink coffee simply because it is the only beverage available.

10. When bones are rendered see that they are cracked and boiled at least six hours. The stock may be used for gravies as well as for soups. A good gravy well blended is a great addition, one with raw flour unblended is pure waste.

11. The servings should be inspected frequently by a commissioned officer to see that the helpings are not too large. Permit as frequent seconds as you wish, but do not put more on at first than the least hungry can clean up.

N. B. — And this will also help — Good news from the Quartermaster Corps : — " Authority is granted for Division Quartermasters to purchase locally, with the consent of the French authorities, such fresh vegetables as may be obtainable at reasonable rates, corresponding reductions on ration returns to be made in the fresh vegetable component of the ration. "

### Pigs and Garbage.

It is recommended, in all camps where it is impracticable to dispose of garbage otherwise than to sell it to the farmers, that pigs be purchased by these camps to eat their own garbage. Eight pounds of garbage will furnish one day's ration for one pig and will produce one pound of fat, worth at present price about 25 cents. This makes the garbage worth 3 cents per pound, a figure which cannot be obtained elsewhere except from a garbage disposal plant.

### Sound advice concerning unsound meat.

" During the past few months, owing to difficulties in transportation, it has been impossible to keep fresh beef frozen until delivered to organizations and some " sweating " or even decomposition has taken place. In many instances whole quarters of such beef have been needlessly condemned on the advice of medical officers, whereas all that was necessary was wiping or washing with salt water or careful trimming of the parts in which decomposition may have taken place. While it is important that unsafe meat be not eaten, much of the meat condemned as decomposed is wholly safe and good. Meat is fortunately now being delivered in excellent condition, but the following suggestions are given to enable officers to save as much as possible if occasions arise.

Whenever a quarter of beef is suspected of taint, first thoroughly wash the quarter with salt and water, examine the exposed surfaces, and if those are tainted cut off such portions as are affected. If the covered surfaces seem to be affected have the butcher remove the covering tissue, taking care not to cut into the flesh. Do not condemn any part of the beef until these preliminary steps have been taken.

To determine whether decay has started within the beef, introduce a probe at the shoulder and hip joints; by the smell at the end of the probe you can determine whether the joints are affected or not. If they are affected, dissect out the bone and trim away the adjacent meat until a sound layer is reached. In no instances is it desirable or necessary to slash the quarter, the object being removal of affected parts with as little waste as possible. To prevent fly-blow make sure that fly eggs are immediately washed off when the beef arrives; these are found usually on the shank. Medical officers should also recognize that there are no absolute tests available for meat spoilage and they are advised to make use of the experience of qualified butchers in the organizations in determining the extent of spoilage.

The following methods are recommended for the best care of frozen beef : — It is better to hang beef in an airy, well-ventilated place out of the direct rays of the sun, rather than to store it in damp, dug-out refrigerators. Meat safes, covered with cheese cloth to exclude flies and with free access of air, will protect the beef for several days if it is wiped as frequently as moisture accumulates on the surface. If it is necessary to retain cut-up beef for more than 24 hours it may be placed in a container and covered with salt and water, but in cutting up beef require the butcher to first remove any tainted outer skin before he cuts into the meat. This avoids the carrying of the decayed portion into the sound meat.

When fresh or frozen meat is received in larger amount than can with advantage be cooked before heat and flies cause deterioration, it is advised that the meat be cut from the bones and be covered with brine in a small barrel cask, or earthenware crock.

### The Clinical and Therapeutic Aspects of Gas Poisoning.

(Extract of lecture delivered by Colonel H. L. Gilchrist, Medical Director of Chemical Warfare Service).

Summary of Clinical Pathology following Phosgene.

In considering the action of lung irritants, the fact must not be lost sight of that the action of any gas depends—First, on the concentration of the gas to which the men are exposed, and Second, on the physical condition of the men.

Two classes of cases can be expected; first the fatal cases and second the serious cases.

1. *Fatal Cases* are confined to the class of men who are subjected to a strong concentration of a strong lung irritant, such as phosgene, without masks. As a result horrible death follows. Death in these cases may follow acute pulmonary oedema, and in certain cases cessation of the respiratory reflexes. As a rule, cases of this kind die in the trenches and never reach the dressing station. The first symptoms of weakness and irregular respiration are immediately followed by pulmonary congestion and asphyxia.

2. *Serious Cases.* The serious cases develop more slowly, as a rule, the patients being exposed to toxic atmosphere during sleep. The first action of the irritant gases is manifested by irritation of the eyes and throat. The alarming features are sudden difficulty in breathing and violent contractions of the throat and thorax. The patient complains of oppressive muscular weakness. If he is walking at the time, he stops, sits down or may perhaps fall prostrate to the ground.

*Pulmonary Symptoms.* These may seem startling. As an example, an officer was exposed to a small quantity of phosgene gas, following which he experienced no serious effect, and retired for the night. On the following morning he arose, feeling in his usual good condition, and he proceeded to his mess tent for lunch. When the meal was over he arose to light a cigarette, fell to the ground with bloody froth flowing from his nostrils and lips, and within a few minutes died of acute pulmonary oedema.

Another example : after an intense bombardment with lung irritant gases a sergeant went forward looking after supplies, etc., following which he descended a small incline and entered his shelter where he was overcome by a paroxysm of acute suffocation. Cyanosis steadily developed, and in less than half an hour he died of asphyxia unaccompanied by expectoration.

The French report several cases where field hospital patients presenting no serious symptoms on arrival, left their beds and were permitted to walk around. Later they were taken with progressive dyspnea, followed by cyanosis and death.

At times the cause of death in these cases is not at all apparent. Patients who, on arrival, manifest no symptoms during auscultation are frequently found, a few hours later, to present asphyxial symptoms, and a further examination shows all the signs of advanced pulmonary oedema.

In the clinical study of gas cases the following stages may be considered : (a) *Complete and acute pulmonary oedema*, followed by death in from a few hours to two days. The rales steadily increase from the onset of symptoms. (b) *Acute, but not fatal, pulmonary oedema.* This stage is followed by lobar or lobular pulmonary congestion especially at the base of the lungs. The large bubbling rale of bronchitis is heard and, little by little, congestion, bronchitis, and ephysema indicate lesions of the respiratory system. (c) *Successive manifestations of pulmonary congestion.* From hour to hour new congestion foci appear. The evolution of congestion may extend through a period of several days. This is especially true for intoxication by Palite and Chlorpicrin. (d) *Simple Bronchitis.* Although simple bronchitis is relatively rare, being confined to light cases, serious affection of the whole bronchial system may bring on advanced dyspnoea and important subjective disturbances.

### Arsenic Gases. New clinical Picture.

The enemy is now using arsenic in the form of (a) ethyldichlorarsine, (b) diphenylchlorarsine, and (c) diphenylcyanarsine; often used with mustard gas (a) can be detected by faint fruity odor; (b) and (c), the latter especially effective in low concentrations, have a pungent smell. The obscure symptoms have misled M. O. s. Be on your guard for :

*Dizziness* and transient unconsciousness, *lethargy* often 12-18 hours (2 cases comatose 3 hours, one with hallucinations), smarting of eyes, nose and throat, no acute pain, mild conjunctivitis. Conjunctival reflex delayed. Pupils dilated in severe cases. Burning sensations over face, no flushing or cyanosis. *Tightness* in chest, slight bronchitis, huskiness and choking, respiratory rate normal. *General* weakness and fatigue with or without nausea and vomiting; low tension, slow pulse; grip weak; legs weak; anesthesia hands and soles of feet decreasing above; present also over upper chest from and back, but not on, abdomen; epi-gastric reflex and ankle colonus present, K. J. absent.

Two or three days later; lethargy decreasing. Pupils dilated react slowly to light, no ocular palsy or nystagnus. Husky voice persists. Elbow jerk greatly exaggerated. Superficial reflexes, epi-gastric, cremasteric, plantar present. Delayed sensations of heat and cold. Others show marked nervous symptoms at Base, but not at Advance Dressing Station. Most cases apathetic, suffer dizziness on standing, lost co-ordination and sense of position; arms and legs weak, gait tottering and shuffling pain in legs when walking but not on pressure over nerves. K. J. rarely normal at first; later sluggish. Improvement usually rapid. In another group, showing at first ordinary symptoms of mustard gas poisoning, some showed indefinite weakness, anesthesia or analgesia (characteristic of chlorarsin) after 8-10 days. Still others ran T 102° F-104 F; P 106-116; R 22, the T returning to normal in 48 hours and possibly due to complications. Headache for 8-12 hours common.

*Pathology* is unknown. Symptoms not due to neuritis, or involvement of root or root zone. Irregularity of symptoms has often raised suspicion of hysteria. Toxic effect central rather than peripheral. Arsenic is said to be found occasionally in urine. Statement needs confirmation.

*Sources.* Contamination of shell hole or other battle area water sources by gases suspected.

### Mustard Burns of Eye.

Treatment of burns of the eye caused by gas (mustard). The eyes should be thoroughly washed out at the earliest possible moment with an alkaline solution such as sodium bicarbonate 1 o/o. While the irritation persists they should be irrigated two to five times daily with a bland solution, normal salt, saturated boric acid or 1 o/o sodium bicarbonate. When the irritation is severe, one or more drops of liquid albolene or olive oil should be instilled after each irrigation. In the severer cases involvement of the corneal epithelium is common and there is a varying degree of photophobia. All such cases should have sol. atropin sulphate 1 o/o instilled from one to three times a day to keep the pupil well dilated. The eyes should be shaded in all cases where conjunctival irritation is evident, but not in the functional variety where the conjunctiva is normal. A secondary conjunctival infection, as evidenced by a a considerable amount of mucopurulent secretion, is not uncommon and is best treated by col. argyrol 20 o/o twice or three times a day. An ophthalmologist should be called in to see the more severe cases.

*Mustard Gas Effect from Smoking Gassed "Durham"* : Pvt. I. W., Co... of the... Infantry, with a shrapnel wound of the right thigh, "rolled his own" from a sack of "Bull Durham" borrowed from a companion who had been gassed. While smoking he remarked upon the gas taint of the tobacco and had to give up the cigarette. Half an hour later definite reddening of the mucous membrane of mouth, lips and adjacent skin was noticeable.

*Gas-Masks of Diphtheria Patients* : The considerable amount of diphtheria reported in the last three weeks direct from combatant troops at the front brings to mind one quite possible source of transmission of mouth and nose secretions, i. e., the gas mask. Although a gas mask is as much a personal belonging as a tooth-brush, or pipe, generosity and emergency know not sanitary laws. Hence it is recommended that the gas masks of men admitted to hospital for anything except battle casualties, i. e. fevers, or infections, be adequately treated by wiping the rubber mouth piece with antiseptic solution and exposing to the direct rays of the sun for two hours, and so attached to the patients' belongings that they may not be issued to another.

*Diphtheria in Gassed Cases.* In a hospital epidemic of 19 clinical cases (June 26-July 10) cultures were made from the throats of 566 persons, patients and personnel, and Schick test performed upon 400 persons. Of the 566 persons "cultured" 71 carriers were found: among 18 medical officers 6, 66 nurses 12, 101 enlisted personnel 3, 32 R. C. and French employes 1, ? patients 49. Susceptibles according to Schick test were immunized. The epidemic evidently had its origin in several cases of "sore throat" among the nurses June 8-10, but none of enough severity to require medical attention, and hence not "cultured".

The incidence of carriers developing among the convalescents from gassing was much higher than among other types of patients. The four wards in which most of the positive patient cultures were found were filled chiefly with severely gassed cases.

### Tuberculosis Again.

The rarer form of peribronchial tuberculosis can best be detected by, (a) Changed tone of the fundamental note at the base of the chest. This note is elicited by heavy percussion and is shorter and higher pitched than normal pulmonary resonance (b) The persistence of indeterminate or bronchial rales limited to the root region of the lung, and heard along the sternum and in the interscapular spaces of the same side. (c) *X-Ray Corroboration* : The sputum of any case held because of suspicious physical or X-ray findings should be examined at least 10 times before being declared non-tuberculous. Regimental surgeons and various specialists in Base Hospitals will have many opportunities to detect early cases of pulmonary tuberculosis.

### Lacerations of the Lids and Eye-Ball.

The special attention of surgeons is called to the necessity of conserving all possible tissue in wounds of the ocular structures. The eyelids are composed of highly differentiated tissue, which cannot be satisfactorily replaced, and loss of even a small amount of skin or of conjunctiva leads to deformity and interference with the function of the eye. Consequently, debridement, if performed at all, should be aimed to preserve every possible bit of tissue which is viable. The same applies to cases where laceration of the eye is so great that evisceration or enucleation must be performed. In this event, as little conjunctiva as is feasible should be sacrificed, in order that the patient may obtain a socket sufficiently large for the wearing of an artificial eye.

The socket should not be packed with gauze after enucleation. A few minutes' pressure is sufficient to control the very moderate hemorrhage. In the very septic cases a small drain, preferably of rubber tissue, may be inserted, but this is seldom necessary.

As far as possible, all ocular surgery should be performed by the specially trained man stationed in each hospital or in one in the neighborhood.

### Control of fleas,

Several sanitary reports mention the flea nuisance as a cause of disturbed sleep, etc. in some of the permanent and temporary barracks in the A. E. F. The following note from the Urologist of the Army Sanitary School may be helpful.

" The flea breeds in the dust and not in the garments of men. It is not, therefore, strictly a body parasite. Stables may be rid of fleas by destroying their breeding places. An earthen floor should be kept sufficiently moist to keep it packed and hard, and occasionally sprinkled with a weak emulsion of crude carbonic acid. A sprinkling with oil will lay the dust more permanently. Where there is a wooden floor the same principles apply, but it should be remembered that there may be dry dust underneath the boards. After a thorough soaking the cracks in the boards may be caulked with oakum to prevent evaporation. The ground outside should receive similar attention. In my experience these procedures may be relied upon to promptly rid a dwelling or stable of fleas ".

### Motor Oil in Sanitation.

No end to the uses of motor by-product. Instead of lampblack and oil, the resourceful sanitarian of the A. E. F. has made this discovery. " Hdqrs. 79 th Division, A. E. F. — We find that cylinder oil after being removed from automobiles that have been cleaned, if mixed with kerosene, is very efficacious and suitable for sanitary purposes, especially for latrines and manure piles. Great quantities of this can be saved in their area and can be used for these purposes. Heretofore it has been thrown away and no use made of it. Would suggest that it be tried out in other areas and if found as efficient as we have, that some means be made to keep this material in the Medical Department for sanitary purposes. Division Sanitary Inspector. "

*Le Gérant :* O. Porée. Paris — Imp. Lahure, 9, rue de Fleurus.

## Figures and Tables

**Figure f1:**
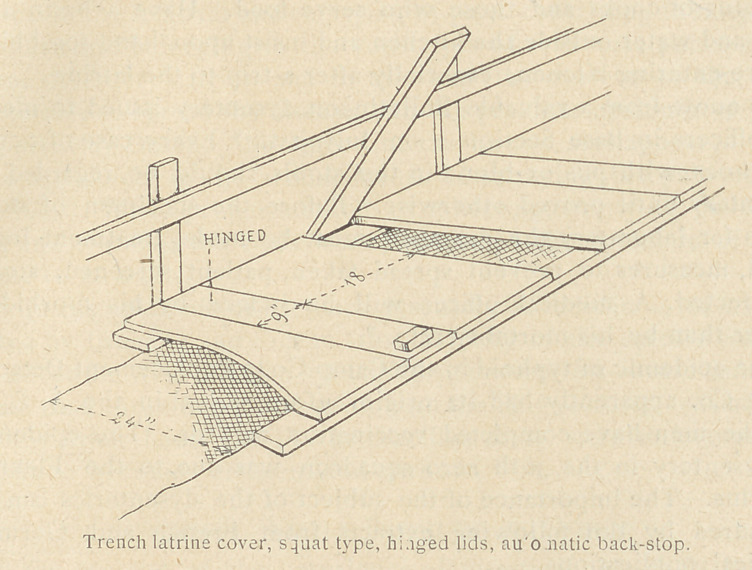


**Figure f2:**
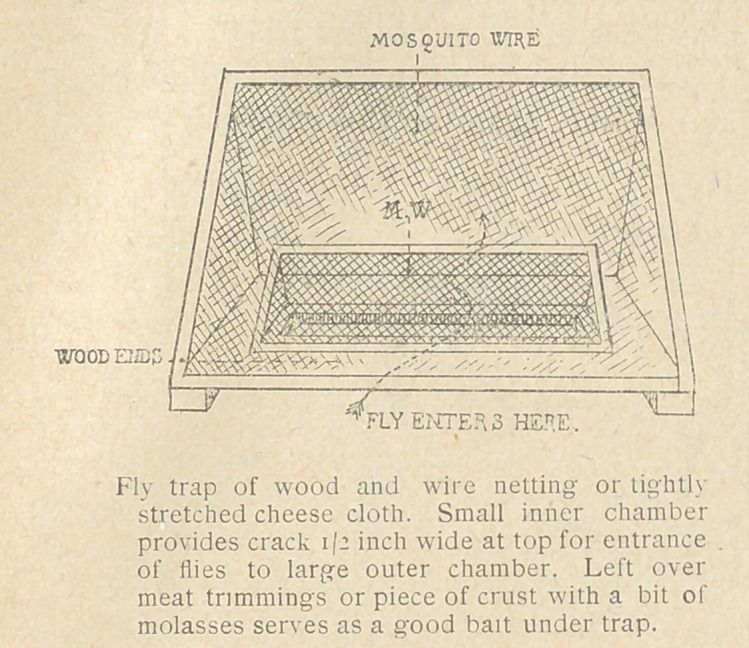


**Figure f3:**
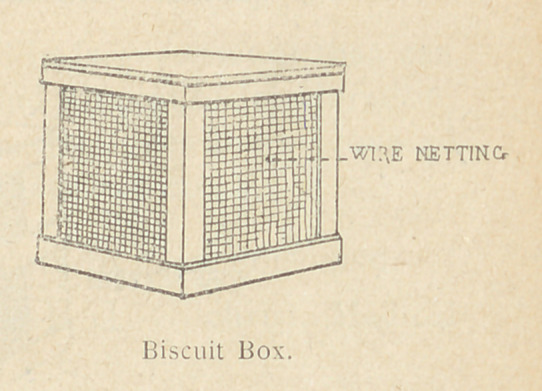


**Figure f4:**
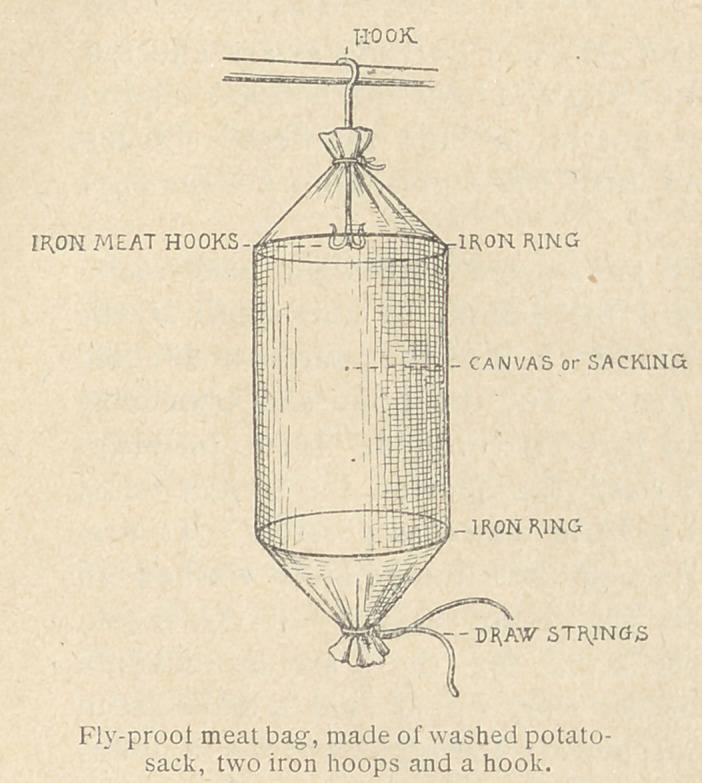


**Figure f5:**
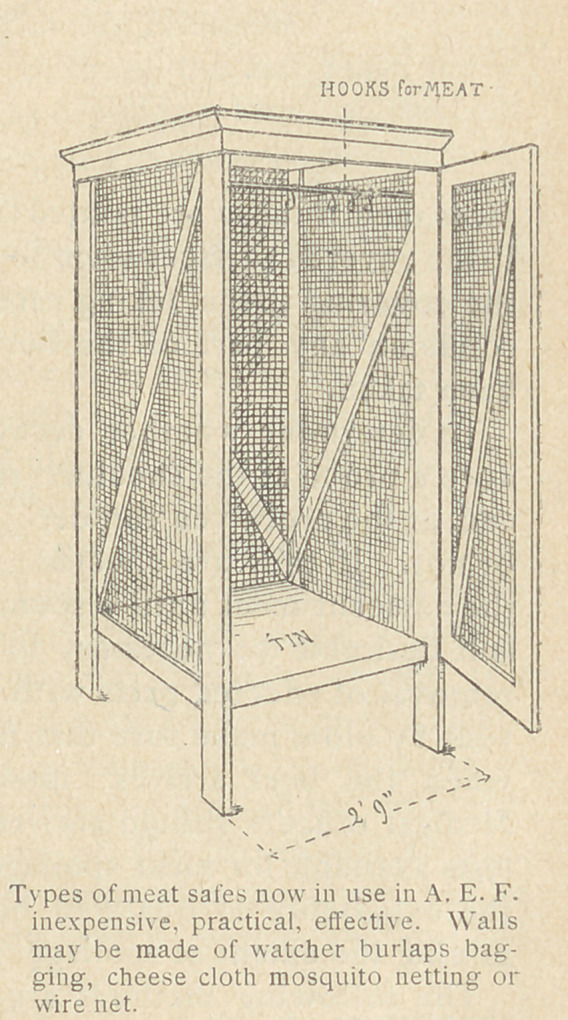


**Figure f6:**